# Central-line team effort: Recognizing insertion-site concerns in nursing homes

**DOI:** 10.1017/ice.2023.165

**Published:** 2023-11

**Authors:** Kristine P. Nguyen, Raveena D. Singh, Raheeb Saavedra, Shruti K. Gohil, John T. Billimek, Steven P. Tam, Karl E. Steinberg, Lori Porter, Susan S. Huang

**Affiliations:** 1 Division of Infectious Diseases, University of California, Irvine School of Medicine, Irvine, California, USA; 2 Department of Family Medicine, University of California, Irvine School of Medicine, Irvine, California, USA; 3 Division of Geriatrics and Gerontology, University of California, Irvine School of Medicine, Irvine, California, USA; 4 Shiley Haynes Institute for Palliative Care, California State University, San Marcos, San Marcos, California, USA; 5 National Association of Health Care Assistants. Carl Junction, Missouri, USA

Central-line–associated bloodstream infections are a major cause of preventable healthcare-associated infections, resulting in an attributable mortality of 12%–25%.^1,2^ Initiatives to combat these infections have been hospital focused; however, efforts in long-term care settings are critically needed. Due to advanced age and comorbidities, nursing home (NH) residents are particularly vulnerable to device-related infections.^3–5^

Standardized monitoring of medical devices is important for proper device care, maintenance, and prevention of infection.^6,7^ Recognizing problematic elements at central-line insertion sites is an important responsibility that requires a team effort for certified nursing assistants (CNAs), licensed vocational nurses (LVNs), and registered nurses (RNs). The common sentiment among NH staff is that central-line care is not encompassed within the CNA role. Although CNAs are not directly responsible for assessing central lines, they are often the first line of defense for noticing and relaying problems because of the greater amount of time they spend with residents. We assessed how well CNAs, LVNs, and RNs were able to identify problematic central-line insertion sites in NHs.

## Methods

A regional Quality Assurance/Performance Improvement (QAPI) program supported by the University of California Irvine was conducted in 8 Orange County, California, NHs to assess attention to central lines (Table 1). Each NH’s Quality Assurance Committee approved the program, and the study was exempt from approval from the institutional review boards. At each NH, a convenience sample of central lines with varying degrees of problematic elements was identified using the Central-Line Insertion Site Assessment (CLISA) score.^6^ Scores ranging from 0 to 3 (increasing score indicates increased severity of local inflammation or infection) were sampled. Study staff used a standardized observation form (online appendix) to evaluate redness, cloudy drainage, peeling dressings, and past-due or undated dressings, and assign a CLISA score.

**Table 1.** Mean Characteristics of 8 Orange County NHs Sampled

Characteristic	Mean (Standard Deviation)
No. of licensed beds	108 (33.8)
Overall CMS star rating^a^	4.1 (1.1)
Mean age	54.0 (11.9)
% Female	78.4 (4.7)
% White	21.3 (16.7)
% Black	29.2 (18.3)
% Asian	2.7 (1.5)
% Hispanic	61.9 (24.3)
% Medicare	20.6 (19.6)
% Medicaid	15.5 (11.0)
% Diabetes	39.1 (9.2)
% Cancer	11.0 (2.1)
% Renal disease	23.9 (3.5)
% Liver disease	2.5 (1.4)

a CMS, Centers for Medicare & Medicaid Services, https://www.medicare.gov/care-compare/.

The CNAs and LVNs/RNs across multiple shifts were shown residents’ devices and were asked to comment on any problems and/or concerns they observed. Staff were also asked open-ended questions about the characteristics of a “picture-perfect line,” the recommended frequency of central-line observations and dressing changes, and the proper response to peeling dressings or signs of inflammation or infection. The percentages of failure to recognize problematic elements were tabulated for CNAs and LVNs/RNs separately.

## Results

**Figure 1.**
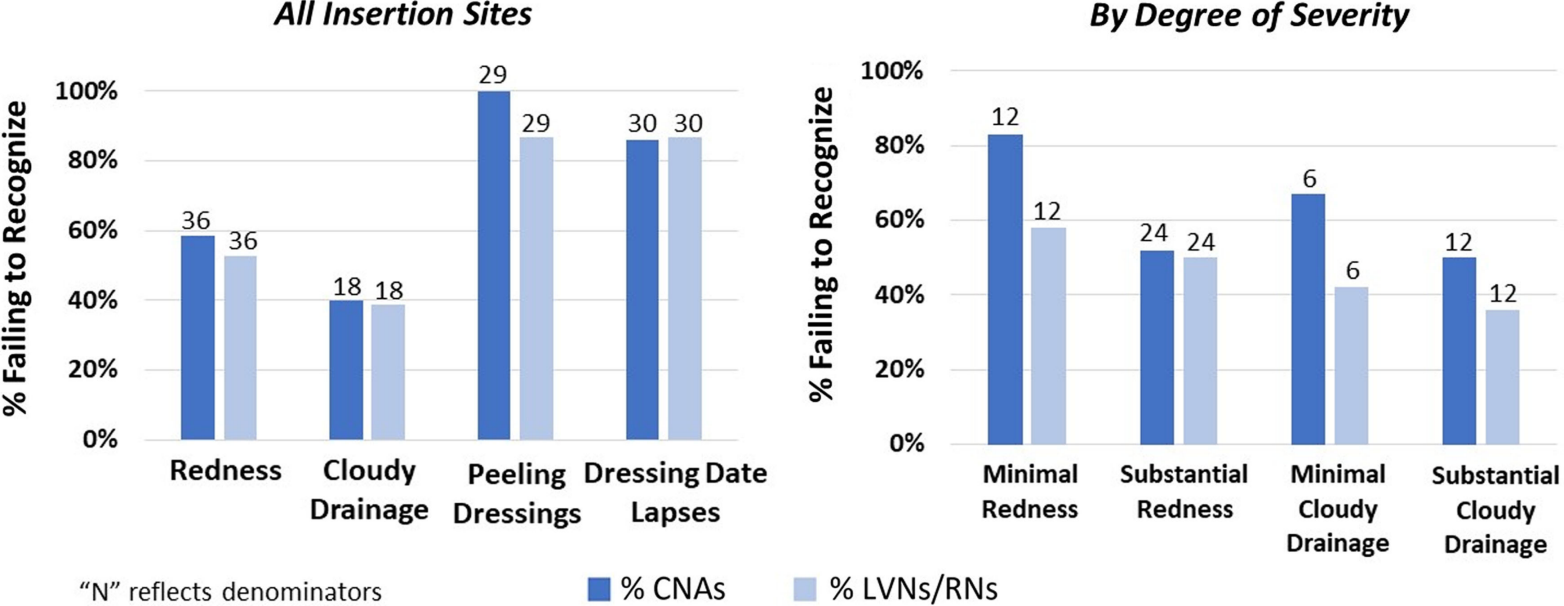
Bar graphs illustrating the frequency of failures to recognize problems at central-line insertion sites. The left panel displays the percentages of failures for all insertion sites observed. Percentages of failure to recognize peeling dressings and lapses in dating dressings were the highest. The right panel stratifies the percentages of failure to recognize redness and cloudy drainage by degree of severity. Recognition of problematic elements improved with more substantial severity.

Across the 8 NHs, 23 central lines were selected and directly observed by up to 6 CNAs and 6 LVNs/RNs each. In total, 50 CNAs (NH range, 3–12) and 50 LVNs/RNs (NH range, 4–9) observed “picture-perfect” lines (N = 7) and lines with redness (N = 8), cloudy drainage (N=5), peeling dressings (N = 7), and inappropriately dated dressings (N=13). Failure to identify problematic elements was frequent (Fig. 1), including failure to identify the following: redness [23 (50%) of 46 CNAs and 19 (53%) of 36 LVNs/RNs], cloudy drainage [14 (40%) of 35 CNAs and 7 (39%) of 18 LVNs/RNs], peeling dressings [26 (100%) of 26 CNAs and 25 (87%) of 29 LVNs/RNs], and inappropriately dated dressings [30 (71%) of 42 CNAs and 13 (68%) of 19 LVNs/RNs].

For both CNAs and LVNs/RNs, recognition of redness and cloudy drainage improved with increased severity. Failure to recognize minimal erythema [10 (83%) of 12 CNAs, 7 (58%) of 12 LVNs/RNs)] was higher than failure to recognize substantial erythema [13 (54%) of 24 CNAs and 12 (50%) of 24 LVNs/RNs] (*P* = .14 for CNAs and *P* = .73 for LVNs/RNs). Similarly, failure to recognize minimal cloudy drainage [4 (67%) of 6 CNAs and 2 (50%) of 4 LVNs/RNs) was higher than failure to recognize substantial cloudy drainage [10 (42%) of 24 CNAs and 5 (36%) of 14 LVNs/RNs] (*P* = .57 for CNAs and *P* = .08 for LVNs/RNs). None of these differences were statistically significant according to the Fisher exact test.

Overall, identification of problematic elements did not vary by whether the staff member was assigned to care for that resident. Descriptions of “picture-perfect lines” and responses to questions about standard care and best practices were uniformly poor, and nursing home staff did not know which elements to mention.

## Discussion

Regular surveillance of central lines is a core infection prevention strategy to promote early detection and response to issues that could increase risk for central-line–associated bloodstream infections. Timely response can mitigate high-risk events.^6,7^ Visual observation of central-line insertion sites is one key way to assure basic practice, but education is needed to ensure that observers are trained to detect relevant problems that should prompt an LVN/RN to take action or prompt a CNA to inform an LVN/RN to assess the line.

Our observations showed that failure to recognize redness, cloudy drainage, peeling dressings, and lapses in dressing change dates was extremely common for CNAs and LVNs/RNs in all NHs surveyed. Furthermore, when stratifying central-line insertion sites by severity (eg, minimal versus substantial erythema and/or cloudy drainage), nursing staff identification of problematic elements improved only minimally for higher-severity insertion sites. Directed training could enable proper recognition and response as well as encourage teamwork.

This study had several limitations. The sample size of NH staff in a localized region was small. Nevertheless, NHs differed in size and proportion of short- to long-stay residents and were variably affiliated with corporations.

Our findings suggest a need for standardized training in NHs to provide clinical staff with the necessary skills to identify central-line problems that commonly arise. First, opportunities exist to train on the key elements of a “picture-perfect line” and ensure a culture of safety and speaking up so that problematic issues are raised and addressed. Second, training is needed to change perceived acceptable thresholds for concern (the line site is “a little red,” the dressing change is “a little late,” there is “a little bit of cloudy drainage”). Providing criteria and expectations can ensure a shared understanding of proper care for both minimal and substantial erythema and cloudy drainage. Lastly, ensuring comprehensive training and expectations about central-line responsibilities (ie, CNAs speak up about potential problems and LVNs/RNs assess and respond) can cultivate improved communication and teamwork between nursing home staff.

## Supplementary material

For supplementary material accompanying this paper visit https://doi.org/10.1017/ice.2023.165. click here to view supplementary material
