# Supplementary material for: Central-line team effort: Recognizing insertion-site concerns in nursing homes
Source: Infect Control Hosp Epidemiol. 2023 Aug 4;44(11):1887–9. doi: 10.1017/ice.2023.165 (PMC10665860; doi:10.1017/ice.2023.165)
Supplement: Supplementary file 1 [file S0899823X23001654sup.zip › S0899823X23001654sup001.pdf]

NH Facility:_______________________________ LVN/RN Initials:________ Resident Room #: ______ Date: ___/____/______

# CENTRAL LINE Maintenance - LVN/RN Skills Assessment:

## Assessment

**Date: ___/___/_____ Staff Initials _____ Resident Room #_____**

**Type of Venous Access: ☐ Midline ☐ Dialysis**

## Observed Practices (non-interview)

### Research staff assessment:

**Admit Date** ........./.........../..........................................

**CLISA Score ☐ 0 ☐ 1 ☐ 2 ☐ 3 ☐ Not Visible** ..........................................

**Any drainage present? ☐ Y ☐ N** ........ **What Type (bloody, serous, pus)**..........................................

**Dressing integrity ☐ All 4 sides adherent ☐ 1 side peeling ☐ 2 or more sides peeling** ..........................................

**Dressing change date clearly marked ☐ Y ☐ N** ..........................................

**Date of dressing change >7 days? ☐ Y ☐ N** ..........................................

**Comments:** ..........................................

### LVN/RN response to viewing line:

| LVN/RN #1: Is this their patient? ☐ Y ☐ N | Concern? | Would Address? | LVN/RN #2: Is this their patient? ☐ Y ☐ N | Concern? | Would Address? | LVN/RN #3: Is this their patient? ☐ Y ☐ N | Concern? | Would Address? |
|---|---|---|---|---|---|---|---|---|
| Redness | ☐ Y ☐ N | ☐ Y ☐ N | Redness | ☐ Y ☐ N | ☐ Y ☐ N | Redness | ☐ Y ☐ N | ☐ Y ☐ N |
| Drainage | ☐ Y ☐ N | ☐ Y ☐ N | Drainage | ☐ Y ☐ N | ☐ Y ☐ N | Drainage | ☐ Y ☐ N | ☐ Y ☐ N |
| Dressing | ☐ Y ☐ N | ☐ Y ☐ N | Dressing | ☐ Y ☐ N | ☐ Y ☐ N | Dressing | ☐ Y ☐ N | ☐ Y ☐ N |
| Change Date | ☐ Y ☐ N | ☐ Y ☐ N | Change Date | ☐ Y ☐ N | ☐ Y ☐ N | Change Date | ☐ Y ☐ N | ☐ Y ☐ N |
| Triggers to Address: ________ ________ ________ | | | Triggers to Address: ________ ________ ________ | | | Triggers to Address: ________ ________ ________ | | |

| LVN/RN #4: Is this their patient? ☐ Y ☐ N | | | LVN/RN #5: Is this their patient? ☐ Y ☐ N | | | LVN/RN #6: Is this their patient? ☐ Y ☐ N | | |
|---|---|---|---|---|---|---|---|---|
| | Concern? | Would Address? | | Concern? | Would Address? | | Concern? | Would Address? |
| Redness | ☐ Y ☐ N | ☐ Y ☐ N | Redness | ☐ Y ☐ N | ☐ Y ☐ N | Redness | ☐ Y ☐ N | ☐ Y ☐ N |
| Drainage | ☐ Y ☐ N | ☐ Y ☐ N | Drainage | ☐ Y ☐ N | ☐ Y ☐ N | Drainage | ☐ Y ☐ N | ☐ Y ☐ N |
| Dressing | ☐ Y ☐ N | ☐ Y ☐ N | Dressing | ☐ Y ☐ N | ☐ Y ☐ N | Dressing | ☐ Y ☐ N | ☐ Y ☐ N |
| Change Date | ☐ Y ☐ N | ☐ Y ☐ N | Change Date | ☐ Y ☐ N | ☐ Y ☐ N | Change Date | ☐ Y ☐ N | ☐ Y ☐ N |
| Triggers to Address: ___ ___ ___ | | | Triggers to Address: ___ ___ ___ | | | Triggers to Address: ___ ___ ___ | | |

## Query to LVN/RN

1. Tell us what a "picture-perfect" line looks like:

   ______________________________________________

   ______________________________________________

2. How often should central line dressings be changed? ☐ Every shift ☐ Daily ☐ Twice a week ☐ Weekly ☐ Every two weeks

3. How often should you assess the central line insertion site? ☐ Every shift ☐ Daily ☐ Twice a week ☐ Weekly ☐ Every two weeks

4. What would you do if you found signs of infection (redness or pus) around the central line insertion site?

   ______________________________________________

5. What would you do if the central line dressing were soiled?

   ______________________________________________

6. What would you do if the central line dressing were peeling?

   ______________________________________________
