# Supplementary material for: Central-line team effort: Recognizing insertion-site concerns in nursing homes
Source: Infect Control Hosp Epidemiol. 2023 Aug 4;44(11):1887–9. doi: 10.1017/ice.2023.165 (PMC10665860; doi:10.1017/ice.2023.165)
Supplement: Supplementary file 1 [file S0899823X23001654sup.zip › S0899823X23001654sup002.pdf]

NH Facility:______________________________ CNA Initials:________ Resident Room #: ______ Date: ___/____/______

CENTRAL LINE Maintenance - CNA Skills Assessment:

Assessment

Date: ___/___/_____ Staff Initials _____ Resident Room #_____

Type of Venous Access: ☐ Midline ☐ Dialysis

Observed Practices (non-interview)

Research staff assessment:

Admit Date ..../.........../..............................

CLISA Score ☐ 0 ☐ 1 ☐ 2 ☐ 3 ☐ Not Visible ....

Any drainage present? ☐ Y ☐ N .... **What Type (bloody, serous, pus)**..............................

Dressing integrity ☐ All 4 sides adherent ☐ 1 side peeling ☐ 2 or more sides peeling ....

Dressing change date clearly marked ☐ Y ☐ N ....

Date of dressing change >7 days? ☐ Y ☐ N ....

Comments: ....

CNA response to viewing line:

CNA #1: Is this their patient? ☐ Y ☐ N	CNA #2: Is this their patient? ☐ Y ☐ N	CNA #3: Is this their patient? ☐ Y ☐ N
Concern? / Would Alert?	Concern? / Would Alert?	Concern? / Would Alert?
Redness ☐ Y ☐ N / ☐ Y ☐ N	Redness ☐ Y ☐ N / ☐ Y ☐ N	Redness ☐ Y ☐ N / ☐ Y ☐ N
Drainage ☐ Y ☐ N / ☐ Y ☐ N	Drainage ☐ Y ☐ N / ☐ Y ☐ N	Drainage ☐ Y ☐ N / ☐ Y ☐ N
Dressing ☐ Y ☐ N / ☐ Y ☐ N	Dressing ☐ Y ☐ N / ☐ Y ☐ N	Dressing ☐ Y ☐ N / ☐ Y ☐ N
Change Date ☐ Y ☐ N / ☐ Y ☐ N	Change Date ☐ Y ☐ N / ☐ Y ☐ N	Change Date ☐ Y ☐ N / ☐ Y ☐ N
Triggers to Alert: ____________ ____________ ____________	Triggers to Alert: ____________ ____________ ____________	Triggers to Alert: ____________ ____________ ____________

CNA #4: Is this their patient? ☐ Y ☐ N

	Concern?	Would Alert?
Redness	☐ Y ☐ N	☐ Y ☐ N
Drainage	☐ Y ☐ N	☐ Y ☐ N
Dressing	☐ Y ☐ N	☐ Y ☐ N
Change Date	☐ Y ☐ N	☐ Y ☐ N

Triggers to Alert:

____

____

____

CNA #5: Is this their patient? ☐ Y ☐ N

	Concern?	Would Alert?
Redness	☐ Y ☐ N	☐ Y ☐ N
Drainage	☐ Y ☐ N	☐ Y ☐ N
Dressing	☐ Y ☐ N	☐ Y ☐ N
Change Date	☐ Y ☐ N	☐ Y ☐ N

Triggers to Alert:

____

____

____

CNA #6: Is this their patient? ☐ Y ☐ N

	Concern?	Would Alert?
Redness	☐ Y ☐ N	☐ Y ☐ N
Drainage	☐ Y ☐ N	☐ Y ☐ N
Dressing	☐ Y ☐ N	☐ Y ☐ N
Change Date	☐ Y ☐ N	☐ Y ☐ N

Triggers to Alert:

____

____

____

Query to Certified Nurse Assistant (CNA)

1. Tell us what a “picture-perfect” line looks like:

   ____

   ____

2. How often should central line dressings be changed? ☐ Every shift ☐ Daily ☐ Twice a week ☐ Weekly ☐ Every two weeks

3. How often should you observe the central line insertion site? ☐ Every shift ☐ Daily ☐ Twice a week ☐ Weekly ☐ Every two weeks

4. What would you do if you found signs of infection (redness or pus) around the central line insertion site?

   ____

5. What would you do if the central line dressing were soiled?

   ____

6. What would you do if the central line dressing were peeling?

   ____
